# Thiazide Use and Fracture Risk: An updated Bayesian Meta-Analysis

**DOI:** 10.1038/s41598-019-56108-4

**Published:** 2019-12-24

**Authors:** Tesfaye Getachew Charkos, Yawen Liu, Lina Jin, Shuman Yang

**Affiliations:** Department of Epidemiology and Biostatistics, School of Public Health, Jilin University, Changchun, 130021 Jilin China

**Keywords:** Fracture repair, Public health, Epidemiology

## Abstract

The association between thiazide use and fracture risk is still controversial. We conducted an updated meta-analysis on the association between thiazide use and fracture risk. We systematically searched PubMed, Embase, and Cochrane library databases for all types of human studies, including observational and experimental studies that were published up until July 2019. We also manually searched the reference lists of relevant studies. The pooled relative risks (RRs) with 95% credible interval (CrI) were calculated using a Bayesian hierarchical random effect model. A total of 19 case-control (N = 496,568 subjects) and 21 cohort studies (N = 4,418,602 subjects) were included in this meta-analysis. The pooled RR for fractures associated with thiazide use was 0.87 (95% CrI: 0.70–0.99) in case-control and 0.95 (95% CrI: 0.85–1.08) in cohort studies. The probabilities that thiazide use reduces any fracture risk by more than 0% were 93% in case-control studies and 72% in cohort studies. Significant heterogeneity was found for both case-control (p < 0.001, I^2^ = 75%) and cohort studies (p < 0.001, I^2^ = 97.2%). Thiazide use was associated with reduced fracture risk in case-control studies, but not in cohort studies. The associations demonstrated in case-control studies might be driven by inherent biases, such as selection bias and recall bias. Thus, thiazide use may not be a protective factor for fractures.

## Introduction

Hypertension and osteoporotic fracture are two major public health problems because they result in a substantial financial burden among the elderly as well as considerable increases in morbidity and mortality^1,2^. Thiazide diuretics are one of the most common types of antihypertension medications^3,4^. There is evidence suggesting that thiazide diuretics reduce urinary calcium excretion^5^, and stimulate osteoblast differentiation and bone mineral formation^6^. Although a previous meta-analysis suggested that thiazide use was associated with reduced fracture risk^7–9^, results of individual studies are still inconsistent, ranging from positive to negative effects^10–19^. In addition, two previous meta-analyses were published over a decade ago^7,8^, and the most recent meta-analysis that was published in 2018 was limited to only prospective cohort studies^9^. Therefore, an updated meta-analysis that is inclusive of all types of study designs is warranted. We conducted a Bayesian meta-analysis on the association between thiazide use and fracture risk as it uses a probabilistic approach to make clinically relevant decisions in the face of uncertainty. For example, using the Bayesian method, we can determine the probability that thiazide use reduces fracture risk by more than 0%, 10% or 20%; this probability is unable to be provided by classical analysis^20^. Therefore, we utilized an advanced methodology in meta-analysis research to address the much controversial relationship between thiazide use and fracture risk that encapsulates all peer-reviewed publications in the field thus far.

## Methods

### Data searching

This study was undertaken according to the Preferred Reporting Items for Systematic Reviews and Meta-Analyses (PRISMA)^21^. We systematically searched PubMed, Embase, and Cochrane library databases for all types of human studies, including observational and experimental studies that were published up until July 2019. The keywords and medical subject headings (MeSH) used for the search were: “thiazide” OR “Sodium Chloride Symporter Inhibitors” AND “Bone fracture” OR “Fracture” OR “Osteoporosis”. We also manually searched the reference lists of relevant studies. Studies were included in the meta-analysis if they met the following criteria: (a) were original human studies; (b) used thiazide as an exposure; (c) had risk estimates for fracture outcome. When more than one study used the same data, we included the most recent and best quality study in our meta-analysis.

### Data extraction and quality assessment

Two investigators (TGC, SY) independently identified and extracted all potential articles for inclusion. Any disagreement between the above two investigators was resolved by discussing it with the third author (YL). The following information was retrieved from each study: first author’s name, year of publication, the percentage of female participants, sample size, fracture outcome, mean age, country, and fracture risk estimates. The Newcastle-Ottawa Scale (NOS) was used to assess the quality of each individual study^22^. Briefly, the NOS score was assessed using the following items: selection, comparability, exposure, and outcome; a NOS score of 7 or higher is considered as high quality^23^.

### Statistical analysis

We synthesized the data using both classical and Bayesian hierarchical random-effects models^24–26^. In classical meta-analysis, we used the DerSimonian-Laird method^27^ to calculate the pooled risk ratio. In the Bayesian model, the risk ratios (RRs) for all the studies were converted into a logarithmic scale (denoted as $\,{\phi }_{i}$). Each ${\phi }_{i}$ was assumed to have a normal distribution with a true, but unknown effect size (${\theta }_{i}$) and known within-study variance (${{\delta }_{i}}^{2}$). The collection of ${\theta }_{i}\,$ across the studies was assumed to have a normal distribution, with unknown mean ($\mu )$ and variance $\,({\tau }^{2}$), where μ was the estimate of the overall log (RR), and ${\tau }^{2}$ was a measure of variation between the studies. The prior information of ${\tau }^{2}$ was assumed to be an inverse gamma distribution (0.001, 0.001). The prior function for μ was assumed equivocal prior; i.e., thiazide use does not affect fracture risk ($\mu$ = 0, variance = 10,000). We also examined the probability that thiazide use reduces fracture risk by more than 0%, 10%, and 20% (i.e., RR < 1.0, 0.9, 0.8). Heterogeneity of the included studies was assessed with Cochran’s Q-statistic test, and inconsistency was quantified by I^2^ statistic^28,29^. Funnel plots were generated to identify potential publication bias using Egger’s test^30^. All analyses were performed by the programs WinBUGS (Version 1.4.3, MRC Biostatistics Unit, Cambridge, UK) and R (Version: 3.4.3; R Foundation for Statistical Computing, Vienna, Austria).

## Results

### Characteristics of studies

**Figure 1:**
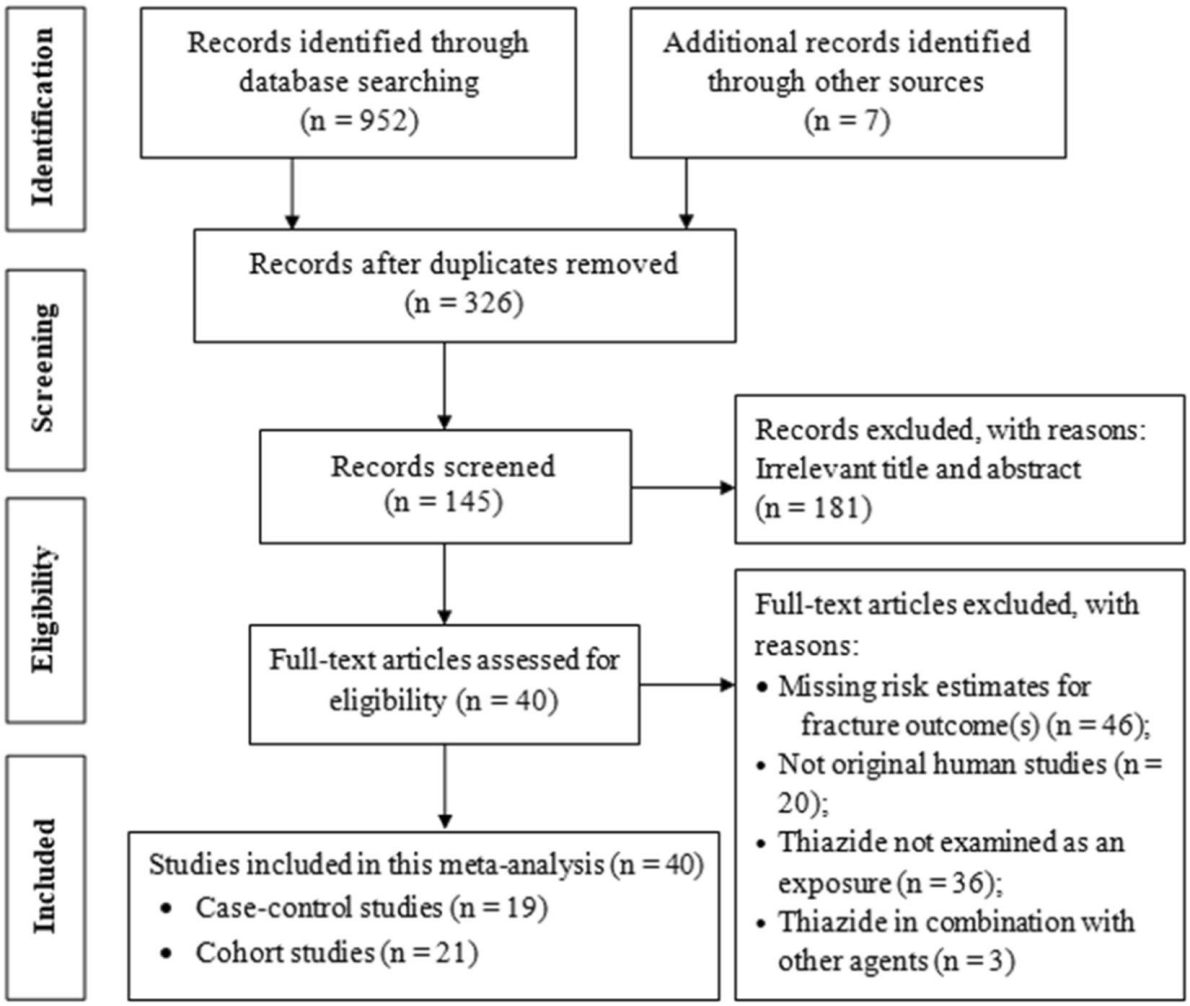
Flow chart for study inclusion and exclusions.

We identified a total of 959 articles from different electronic databases and other sources. Of these, 633 duplicate articles and 181 irrelevance articles were excluded after reading the title or abstract. Finally, 19 case-control studies and 21 cohort studies were met for inclusion in this meta-analysis (Fig. 1). A majority (72.5%) of the included studies were considered as high quality based on NOS standards (Table 1). In the case-control studies, approximately 79% of the participants (Total sample size = 496,568) were female; the average participant age in the case-control studies was 72 years old. Approximately 63% of the subjects (Total sample size = 4,418,602) were female in cohort studies. The average participant age in the cohort studies was 73 years old.

**Table 1:** Descriptive characteristics for included studies.

Author(s)	Percentage of females		Sample size	Fracture outcome	Mean age^a^	Country	NOS score
**Case-control study**							
Rashiq^16^		49	306	Hip fracture	79/78	UK	7
Ray^38^		74	6137	Hip fracture	NA	Canadian	7
Stevens^39^		79	307	Hip facture	79/77	UK	5
Heidrich^40^		76	924	Hip fracture	NA	USA	7
Felson^41^		100	848	Hip fracture	77/78	UK	9
Jensen^42^		83	400	Hip fracture	80/80	Denmark	7
Cumming^43^		NA	416	Hip fracture	65/65	Australia	9
Herings^44^		74.9	772	Hip fracture	78/78	Netherland	8
Barengolts^45^		NA	436	Hip fracture	70/70	USA	6
Weiland^46^		100	725	Hip fracture	73/73	Germany	8
Wang^47^		84	6110	Hip fracture	84/84	USA	7
Luetters^48^		77	3286	Foot fracture	59/65	USA	7
Schlienger^49^		NA	151420	Any fracture	NA	UK	8
Kelsey^50^		78	2594	Pelvis	NA	USA	5
Rejnmark^17^		65	258810	Any/hip/vertebral	66/66	Denmark	9
Kelsey^51^		77	2578	Tibia, fibula	45/45	USA	7
Peters^52^		60	3845	Any fracture	84/84	USA	4
Berry^53^		NA	56,416	Hip fracture	NA	UK	8
Vecchis^54^		100	238	Vertebral	69	Italy	4
**Cohort study**							
Cauley^55^		100	9704	Any/hip/humerus	72	USA	9
Cumming^56^		100	9516	Hip fracture	NA	Australia	8
Nguyen^57^		0	820	Any/hip/vertebral	NA	Australia	6
Guo^58^		74	1608	Hip fracture	82	Sweden	7
Feskanich^59^		100	83728	Any/hip fracture	NA	USA	7
Schoofs^60^		NA	7891	Hip fracture	NA	Netherland	7
Solomon^19^		80	376061	Any/hip/humerus	80	USA	8
Butt^5^		81	1463	Hip fracture	81	Canadian	8
LaCroix^61^		61	9518	Hip fracture	74	UK	8
Chow^62^		66.	439	Any fracture	71	China	7
Carbone^63^		0	6969	Vertebral fracture	59	USA	4
Bokrantz^64^		55	60893	Any fracture	66	Sweden	7
Ruths^18^		56	906422	Hip fracture	73	Norway	8
Kruse^11^		NA	1123670	Any/hip/vertebral	69	Denmark	7
Paik^14^		100	55780	Vertebral fracture	67	UK	3
Chen^65^		56	1144	Any fracture	77	Taiwan	8
Puttnam^15^		43	22180	Hip/Pelvic	70	USA	7
Torstensson^66^		54	1586554	Any fracture	75	Denmark	5
Lin^12^		42	7470	Hip fracture	NA	Taiwan	5
Kim^10^		59	137304	Any fracture	73	South Korea	7
Lin^13^		42	9468	Vertebral fracture	NA	Taiwan	6

^a^Mean ages are reported separately for case-control studies (case/control).

Abbreviations: NA: Not available; NOS: Newcastle Ottawa Scale.

### Thiazide use and fracture risk in case-control studies

**Figure 2:**
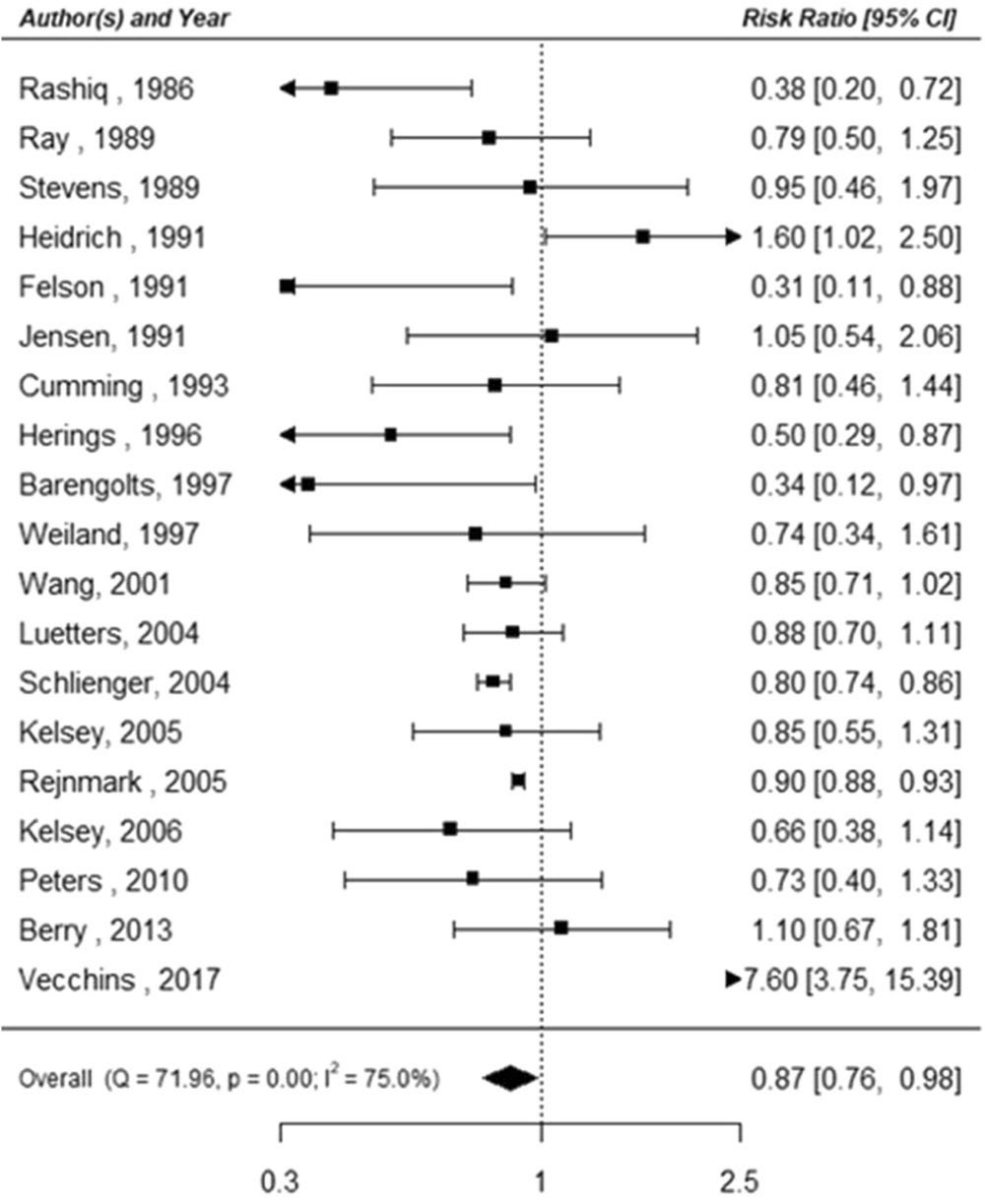
Association between thiazide use and fracture risk for case-control studies analyzed using the classical meta-analysis approach.

In the classical meta-analysis of case-control studies, we found a negative association between thiazide use and fracture risk (Risk ratio (RR): 0.87, 95% confidence interval (CI): 0.76–0.98). We observed moderate heterogeneity between studies (p < 0.001, I^2^ = 75%; Fig. 2). In the Bayesian analysis, the pooled RR for fractures associated with thiazide use was 0.87 (95% credible interval (CrI) 0.70–0.99). The probabilities that thiazide use reduces fracture risk by more than 0%, 10%, and 20% were 93%, 66%, and 23%, respectively (Table 2).

**Table 2:** Bayesian Meta-Analysis: Association between thiazide use and fracture risk.

Subgroup	No. of studies	RR (95% CrI)	Probability (%) that risk ratio		
			≤1.0	≤0.9	≤0.8
Case-control studies	19	0.87 (0.70, 0.99)	0.93	0.66	0.23
Cohort studies	21	0.95 (0.81, 1.08)	0.72	0.23	0.02

Abbreviations: RR: risk ratio; CrI: Credible interval.

### Thiazide use and fracture risk in cohort studies

**Figure 3:**
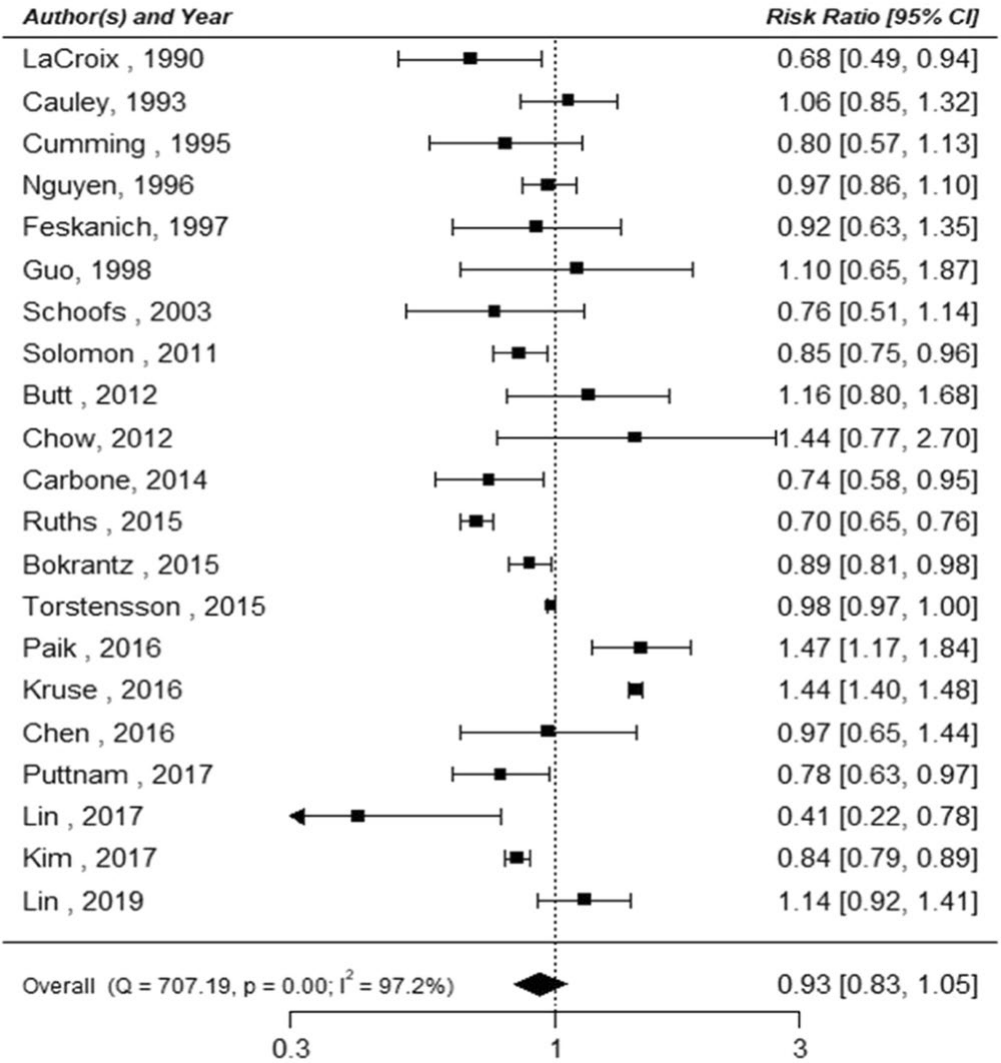
Association between thiazide use and fracture risk for cohort studies analyzed using the classical meta-analysis approach.

In the classical meta-analysis of cohort studies, there was no significant association between thiazide use and fracture risk (RR: 0.93, 95% CI: 0.83–1.05). The heterogeneity between studies was significant (p < 0.001, I^2^ = 97.2%; Fig. 3). In the Bayesian analysis, the pooled RR for fractures associated with thiazide use was 0.95 (95% CrI: 0.85–1.08). The probabilities that thiazide use reduces fracture risk by more than 0%, 10%, and 20% were 72%, 23%, and 2%, respectively (Table 2).

### Publication bias

**Figure 4:**
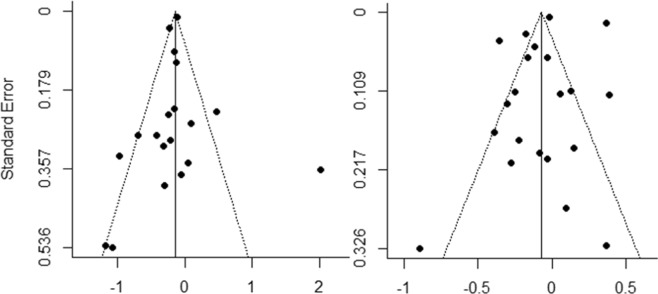
Funnel plot of risk ratio versus standard error for the association between thiazide use and fracture risk. (**a**) For case-control studies. (**b**) For cohort studies.

The funnel plot of risk ratio versus standard error for the association between thiazide use and fracture risk was shown in Fig. 4. No significant publication bias was observed for both case-control studies (Egger’s test: p = 0.65; Fig. 4a) and cohort studies (Egger’s test: p = 0.52; Fig. 4b).

## Discussion

This meta-analysis provides evidence to support that thiazide exposure is associated with a 13% reduction of fracture risk in case-control studies. However, while an inverse association was noted in cohort studies, it failed to reach statistical significance.

Our findings were partly comparable with the effect shown in the previous two meta-analyses reported by Wiens *et al*.^8^ and Xiao *et al*.^9^; both studies suggested that thiazide was associated with the reduction of any fracture risk by 14%. However, to the best of our knowledge, our meta-analysis is the first to distinguish a difference in the relationship between thiazide use and fracture risk by study design. We found that there is a null relationship between thiazide use and fracture risk in cohort studies. A recently published meta-analysis also suggested that the effect of thiazide use on fracture risk was weaker in cohort studies^9^. Although the results from the Bayesian meta-analysis were consistent with that generated from the classical meta-analysis approach, the Bayesian meta-analysis provides additional regarding the probabilities that thiazide use reduces fracture risk by certain percentages. Such information is useful for making clinically relevant decisions about the use of thiazides, and cannot be obtained using the traditional meta-analysis methodology.

The controversial relationship between thiazide diuretics and fractures involves conflicting mechanisms. On the one hand, thiazide could exert beneficial effects on the bone via decreasing urinary calcium excretion by 25–40%^31,32^. In addition, thiazides are associated with an increased level of metabolic alkalosis, which is an inhibitor of bone resorption^33,34^. On the other hand, thiazides diuretics could induce hyponatremia, which has a negative impact on the metabolism and integrity of the bone^35,36^. In addition, thiazide induced-hyponatremia could have harmful neurological side effects, such as gait disturbances and imbalance, which leads to an increased risk of falls and fractures^37^.

This meta-analysis has several limitations. First, due to the absence of relevant experimental studies in humans, our meta-analysis included only observational studies. A meta-analysis based on observational studies cannot make causal inferences about thiazide use and fracture risk. Second, we observed considerable heterogeneity between individual studies, which might bias our results. Lastly, due to insufficient data from individual studies, we did not evaluate the effect of dose and duration of thiazide use on bone fractures.

In conclusion, this meta-analysis included 19 case-control and 21 cohort studies to examine the relationship between thiazide use and fracture risk. Our results suggest that thiazide use was associated with reduced fracture risk in case-control studies, but not in cohort studies. The associations demonstrated in case-control studies might be driven by inherent biases such as selection bias and recall bias. Thus, thiazide use may not be a protective factor for fractures. Randomized clinical trials are still warranted to confirm our findings.

## Data Availability

The data analyzed for the current study are all available from the published individual studies.
